# First record of the genus *Pseudamblyopus* (Coleoptera, Erotylidae) in China, with description of a new species

**DOI:** 10.3897/zookeys.1040.59547

**Published:** 2021-05-26

**Authors:** Jing Liu, Weicheng Lu, Haoming Zang, Jing Li

**Affiliations:** 1 College of Plant Protection, Hebei Agricultural University, Baoding 071001, China Hebei Agricultural University Baoding China

**Keywords:** The genus *Pseudamblyopus* Araki, 1941 is reported from China for the first time. *Pseudamblyopus sinicus* Liu & Li, **sp. nov.** is described and illustrated, and one species previously placed in *Scelidopetalon* Delkeskamp, 1957 is transferred to *Pseudamblyopus* resulting in the following new combination: *Pseudamblyopus varicolor* (Arrow, 1925). A key to all known species of *Pseudamblyopus* is given. Key, new combination, new record genus, taxonomy

## Introduction

The family Erotylidae currently contains approximately 3500 species in 258 genera worldwide, with the highest diversity in South America, Africa, and Asia (Leschen et al. 2010). The tribe Tritomini Curtis, 1834 is the largest tribe in the subfamily Erotylinae sensu stricto, one of six subfamilies in the family Erotylidae, with 92 genera and approximately 1200 species (Leschen et al. 2010; Skelley and Powell 2018). The genus *Pseudamblyopus* was erected by Araki in 1941 with *Amblyopus palmipes* Lewis, 1889 as its type species. Until now, only two species, *Pseudamblyopus similis* (Lewis, 1887) in Japan and Russia and *P. palmipes* (Lewis, 1889) in Japan, have been reported (Chûjô and Chûjô 1990; Wegrzynowicz 2007). In the Fauna Japonica, Chûjô (1969) described these species in detail. The genus *Pseudamblyopus* is similar to the genera *Amblyopus* Lacordaire, 1842 and *Scelidopetalon* Delkeskamp, 1957. Both *Pseudamblyopus* and *Scelidopetalon* can be distinguished from *Amblyopus* by their small and finely facetted eyes (Araki 1941; Dai and Zhao 2013). While most species of *Scelidopetalon* differ from those of *Pseudamblyopus* by having the antennomere XI much wider than long, in *Scelidopetalon varicolor* (Arrow, 1925) and other species of *Pseudamblyopus* antennomere XI is almost as long as wide or only slightly wider (Dai and Zhao 2013). The ratio of width to length of the antennomere XI of all species in *Pseudamblyopus* is 1.2–1.67:1, while *Scelidopetalon* is 2.1–2.5:1 except *S. varicolor*. The antennomere XI of *S. varicolor* is 1.5× as wide as long, which is the same as that in other representatives of *Pseudamblyopus*. So, *S. varicolor* (Arrow, 1925) is here transferred to *Pseudamblyopus* Araki, 1941 [*Pseudamblyopus varicolor* (Arrow, 1925), comb. nov.]. *Pseudamblyopus sinicus* sp. nov. from Guangdong Province, China, is described and illustrated. Before this study, there was no record of *Pseudamblyopus* in China.

## Materials and methods

The abdominal segments and the genitalia were detached from the body after softening in hot water. Male and female genitalia were placed in 5% NaOH boiling solution for 5 min and then cleaned with distilled water. Morphological characters were illustrated using a Nikon SMZ800N stereomicroscope and modified with Adobe Photoshop CS6.0. Habitus photographs were taken with an Olympus E-M5II camera. Terminology for major structures follow Lawrence et al. (2010, 2011). We have examined all specimens of *Scelidopetalon* and *Pseudamblyopus* deposited in the Museum of Hebei University (**MHBU**), Natural History Museum (**NHML**), and Department of Biology, Shanghai Normal University (**SHNU**).

### Species of *Scelidopetalon* and *Pseudamblyopus* examined in the current studies

*Scelidopetalon instabilis* (Gorham, 1896) [*Petaloscelis*] (Burma, Vietnam) from the **NHML**.

*Scelidopetalon similis* (Arrow, 1925) [*Petaloscelis*] (Assam Valley) from the **NHML**.

*Scelidopetalon solidus* (Arrow, 1925) [*Petaloscelis*] (India) from the **NHML**.

*Scelidopetalon fasciatus* (Arrow, 1926) [*Petaloscelis*] (Sumatra, N. Borneo) from the **NHML**.

*Scelidopetalon arrowi* Delkeskamp, 1957 (Singapore) from the **NHML**.

*Scelidopetalon monommoides* (Arrow, 1917) [*Petaloscelis*] (Cameroon) from the **NHML**.

*Scelidopetalon biwenxuani* Dai & Zhao, 2013 (China) from the **SHNU**.

*Pseudamblyopus varicolor* (Arrow, 1925), comb. nov. [*Petaloscelis*] (India) from the **NHML**.

*Pseudamblyopus palmipes* (Lewis, 1889) [*Amblyopus*] (Japan) from the **NHML**.

*Pseudamblyopus similis* (Lewis, 1887) [*Amblyopus*] (Russia, Japan) from the **NHML**.

## Taxonomy

### Genus Pseudamblyopus

Taxon classificationAnimaliaColeopteraErotylidae

Araki, 1941

37D4935D-4285-5007-9969-44D3CCB9E4EC

#### Type species.

*Amblyopus palmipes* Lewis, 1889.

#### Diagnosis.

Body small to medium-sized, oval to elongate oval, distinctly convex dorsally. ***Head*** with a pair of stridulatory files on the occipital region; lacinia without teeth at apex; terminal maxillary palpomere nearly triangular to semicircular; mentum much longer than wide, sharply and triangularly ridged on its surface; terminal labial palpomere elongate but not dilated terminally. Compound eye small and finely facetted; antennae rather short, antennomere III nearly equal in length to antennomere IV and V combined; antennal club compactly articulated, antennomere XI irregularly rounded, almost as long as wide, and much narrower than preceding segment. ***Pronotum*** approximately twice as wide at the base as long. The base of pronotum narrower than the base of elytra. ***Elytra*** convex, with eight regular rows of fine punctures on each elytron located in bottom of longitudinal furrows (striae). ***Prosternum*** rather short, prosternal process wide, widened posteriorly, markedly emarginate at its posterior border. ***Prosternum*** with prosternal lines, metaventrite with postmesocoxal lines and basal abdominal ventrite with postmetacoxal lines.

***Legs*** rather short and robust; tibiae markedly expanded terminally.

***Sexual dimorphism*:** male with legs more robust than in female, with extended and more dilated protarsi.

#### Distribution.

Japan (Hokkaido, Honshu, Shikoku, Kyushu), China (Guangdong), India (Nilgiri Hills), Russia (Far East).

### Key to species of the genus *Pseudamblyopus*

Partly based on Arrow (1925) and Dai and Zhao (2013).

**Table:** 

1	Elytra with indistinct reddish patch at base. Body length: 4.0–5.0 mm. Distribution: India (Nilgiri Hills)	***Pseudamblyopus varicolor***
–	Elytra without reddish patch at base	**2**
2	Pronotum with two colors, black semicircular spot at the basal border of pronotum. Body length: 4.6–5.1 mm. Distribution: China (Guangdong)	***Pseudamblyopus sinicus* sp. nov.**
–	Pronotum uniformly reddish brown	**3**
3	Leg reddish-brown. Body length: 3.5–5.5 mm. Distribution: Japan (Hokkaido. Honshu, Shikoku) and Russia (Far East)	***Pseudamblyopus similis***
–	Leg black. Body length: 4.5–6.5 mm. Distribution: Japan (Honshu, Shikoku, Kyushu)	***Pseudamblyopus palmipes***

### Pseudamblyopus sinicus

Taxon classificationAnimaliaColeopteraErotylidae

Liu & Li sp. nov.

0742B2BD-9705-57BB-9786-C59347D20359

http://zoobank.org/212740A3-1C5B-46EE-B903-071389B9342B

Figures 1 , 2–14

#### Material examined.

***Holotype*** (MHBU), ♂: China; Guangdong Province, Nankun Mountain; 23°09'1.47"N, 113°20'42.70"E; VII/30/2010; Hao Yu Liu, leg. ***Paratype*** (MHBU), (1 ♀). 1 ♀: same data as holotype.

#### Diagnosis.

Body oval, distinctly convex dorsally, smooth and glossy; general color reddish brown. Pronotum with basal border of pronotum slightly infuscate and one black semicircular spot at basal middle, scutellar shield, elytra, basal border of prosternum, mesoventrite, metaventrite and abdominal ventrites black. Antennomere III slightly longer than antennomeres IV and V combined. Terminal maxillary palpomere triangular, about 1.8× as wide as long. Pronotum nearly trapezoidal, convex dorsally; 1.6× as wide as long, finely and closely punctured. Tibiae strongly expanding at apex.

**Figure 1.**
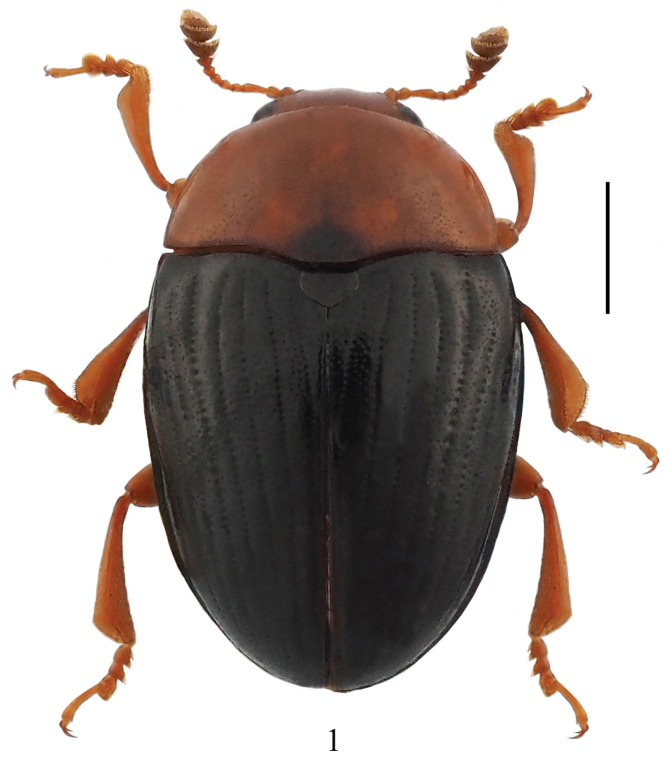
Dorsal habitus of *Pseudamblyopus sinicus* sp. nov. Scale bar: 1.00 mm.

#### Comparison.

This species is similar to *Pseudamblyopus similis* due to the shape and color of the body. The new species can be identified using the above key to species. It is distinguished by its pronotum having the black semicircular spot at the basal border; scutellar shield subangulate posteriorly; basal border of prosternum, mesoventrite, metaventrite, and abdominal ventrites black. In contrast to the new species, *P. similis* has its pronotum without black spot; scutellar shield rounded posteriorly; basal border of prosternum, mesoventrite, metaventrite, and abdominal ventrites reddish brown.

#### Description.

Body length: 4.6–5.1, width: 3.0–3.2mm. Body oval, distinctly convex dorsally, smooth and glossy, general color reddish brown, basal border of pronotum slightly infuscated, with one black semicircular spot at basal middle, scutellar shield, elytra and basal border of prosternum, mesoventrite, metaventrite and abdominal ventrites black (Fig. 1).

**Figures 2–14.**
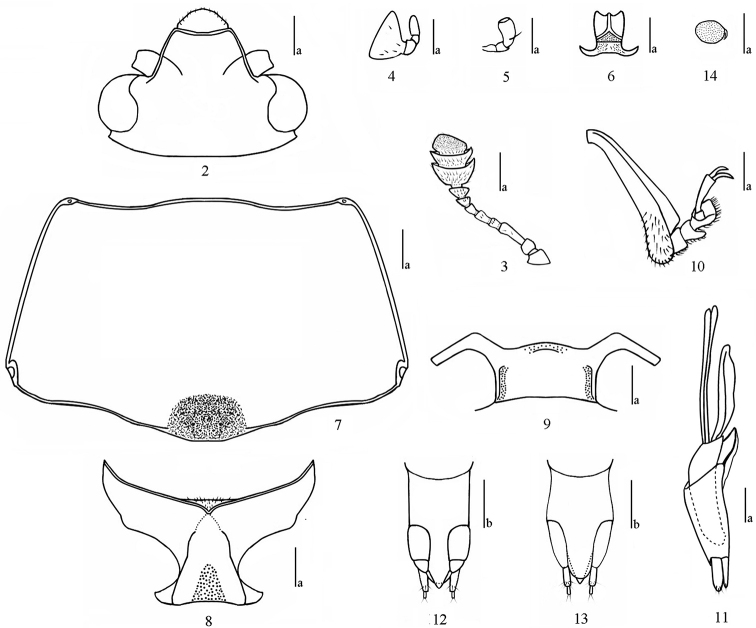
*Pseudamblyopus sinicus* sp. nov. **2** head **3** antenna **4** maxillary palpus **5** labial palpus **6** mentum and submentum **7** pronotum **8** prosternum **9** mesoventrite **10** protibia and protarsus **11** aedeagus, lateral view **12, 13** ovipositor, ventral, and dorsal views **14** female spermatheca. Scale bars: 0.25 mm (**a**), 0.5 mm (**b**).

***Head*** (Fig. 2) large, with and dense punctures. Labrum semicircular, with golden pubescence at anterior border; mandibles protruding; clypeus with narrow border and lateral continuations nearly reaching eyes, feebly emarginate in middle, with one fovea on each side of base; frontoclypeal suture incomplete. Compound eye large, moderately prominent, finely faceted; interocular distance 0.62× width of head. Antennae (Fig. 3) short, extending to basal half of pronotum, with golden setae; antennomere I (scape) rather large; antennomere II subtriangular; antennomere III slightly longer than antennomere IV and V combined; antennomeres VI–VIII nearly equal; antennomere VII and VIII somewhat expanding; antennomeres IX and X bowl-shaped and much wider than long, antennomere XI (width: length = 1.67:1) irregularly rounded; relative lengths of antennomeres II–XI: 1.1: 2.9: 1.1: 1.4: 1.0: 1.0: 1.1: 2.0: 2.3: 2.3. Maxillary terminal palpomere (Fig. 4) triangular, sides rounded, nearly 1.8× as wide as long. Labial terminal palpomere (Fig. 5) subcylindrical. Mentum (Fig. 6) with subtriangular plate, both sides with marginal border, middle area depressed; submentum (Fig. 6) finely and sparsely punctured, with few setae.

***Pronotum*** (Fig. 7) nearly trapezoidal, widest at basal, convex dorsally; finely and densely punctured. Anterior margin shallowly bisinuate, with narrow and complete marginal border; lateral margins broadly rounded, with expressed border; basal margin weakly bisinuate, with border at both sides, but not along median antescullar part. Anterior and posterior angles rounded, each with one pore. ***Prosternum*** (Fig. 8) almost impunctate laterally, with fine and sparse punctures medially, with golden setae; anterior border produced to short point in middle, with narrow and complete marginal border; prosternal process with subtriangular depression at apical emargination, surface with golden pubescence; prosternal lines extending anterior margin of procoxal cavities. ***Scutellar shield*** large, subpentagonal, transverse, with fine and dense punctures, subangulate posteriorly. ***Elytra*** with eight striae bearing distinct and rather coarse punctures.

***Mesoventrite*** (Fig. 9) wide, each side with one shallow depression. ***Metaventrite*** coarsely punctured at sides and finely punctured in middle; postmesocoxal lines long, extenging to basal 3/5 of metaventrite. ***Abdomen*** with coarse and dense punctures laterally and slightly finer ones medially; postmetacoxal lines extending to basal 3/4 of ventrite I.

***Legs*** short, femora dilated medially; tibiae (Fig. 10) subtriangular.

***Male genitalia*** (Fig. 11): median lobe weakly curved, gradually narrow from base to apex; median strut long, with apex dilated and about as long as median lobe. Flagellum present and without sclerite at base.

***Female genitalia*** (Figs 12, 13): ovipositor with narrow styli at apex of coxite, covered with long setae; spermatheca (Fig. 14) nearly oval.

#### Distribution.

China (Guangdong Province).

#### Etymology.

The species epithet means Chinese.

## Supplementary Material

XML Treatment for Pseudamblyopus

XML Treatment for Pseudamblyopus sinicus
